# Seasonal variation in the Canastra cheese mycobiota

**DOI:** 10.3389/fmicb.2022.1076672

**Published:** 2023-02-03

**Authors:** José Guilherme Prado Martin, João Marcos Maia Silva, Isabel Cristina da Rocha César, Meiriele da Silva, Samara Aparecida Santana, Tomás Gomes Reis Veloso, Jonas Guimarães e Silva, Celia Lucia de Luces Fortes Ferreira, John Leech, Paul D. Cotter

**Affiliations:** ^1^Microbiology of Fermented Products Laboratory (FERMICRO), Department of Microbiology, Universidade Federal de Viçosa, Viçosa, Brazil; ^2^Laboratory of Mycorrhizae (LAMIC), Department of Microbiology, Universidade Federal de Viçosa (UFV), Viçosa, Brazil; ^3^Instituto Federal de Minas Gerais (IFMG), Bambuí, Brazil; ^4^Department of Food Technology, Universidade Federal de Viçosa (UFV), Viçosa, Brazil; ^5^Teagasc Food Research Centre, Moorepark, Fermoy, Ireland; ^6^APC Microbiome Ireland and VistaMilk, Fermoy, Ireland

**Keywords:** fungi, mold, yeast, artisanal minas cheese, artisanal cheese

## Abstract

Canastra cheese is the most well-known artisanal cheese produced in Brazil. Although its production includes a step to remove fungi from the cheese surface, in recent years some cheesemakers have preserved the autochthonous fungi grown during ripening due to an interest in the sensory characteristics attributed to these microorganisms. In this work, the mycobiota of artisanal cheeses produced in the Canastra region was characterized based on ITS marker gene analysis. A total of 96 artisanal cheeses from 16 different farms across 9 cities were collected during two different periods (dry and wet seasons). The Canastra cheese mycobiota was significantly impacted by the season, the city of production and the farm but altitude did not affect the fungal community of the cheeses analyzed. *Debaryomyces prosopidis* was most abundant in the majority of samples across both seasons. During the wet season, *Trichosporon asahii, Kluyveromyces lactis* and *Fusarium solani* were the next most abundant species, followed by *Torulaspora delbrueckii* and *Acremonium citrinum.* These results highlight the importance of manufacturing practices and seasonality on the fungal composition of Canastra cheeses. These insights are particularly important in light of recent new regulation in Brazil, removing previous obstacles for surface fungi to persist on cheese. These new regulations will allow new approaches to cheese production, and ultimately, novel products.

## 1. Introduction

Artisanal Minas Cheese (AMC), produced in the state of Minas Gerais, is one of the most famous artisanal cheeses in Brazil, whose production method was recognized as part of the Brazilian cultural heritage by the Instituto Celia Lucia de Luces Fortes Ferreira Patrimônio Histórico Nacional (Instituto do Patrimônio Histórico e Artístico Nacional [IPHAN], 2014). AMC must be produced with raw whole cow’s milk, an endogenous ferment (popularly known as “pingo”), rennet and salt. It must have particular characteristics and be ripened according to the period determined on the basis of scientific studies, which can vary according to the producing region (Diário Oficial do Estado de Minas Gerais, 2020). Currently, it is estimated that around 30,000 producers are involved in the production of AMC. In the traditionally characterized and recognized micro-regions, there are about 9,000 producers, distributed among the micro-regions of Araxá, Campo das Vertentes, Canastra, Cerrado, Serra do Salitre, Serro, Triângulo Mineiro and, more recently, Serras da Ibitipoca (Instituto Mineiro de Agropecuária [IMA], 2020; Empresa Brasileira de Pesquisa Agropecuária [EMBRAPA], 2019).

The quality of the cheeses produced in the Canastra region has been recognized in national and international awards (Mondial du Fromage, 2021; Prêmio Queijo Brasil, 2021). Canastra cheeses can be produced in 9 different municipalities in the Canastra micro-region: Bambuí, Córrego D’Anta, Delfinópolis, Medeiros, Piumhi, São Roque de Minas, São Batista da Glória, Vargem Bonita and Tapiraí (Instituto Mineiro de Agropecuária [IMA], 2018). By law, Canastra cheeses must be matured for at least 14 days (Instituto Mineiro de Agropecuária [IMA], 2021) and have a semi-hard consistency with a tendency to a soft, compact texture, slightly acidic and non-spicy flavor, white-yellowish colour and a thin and yellowish rind, without cracks (Empresa de Assistência Técnica e Extensão Rural de Minas Gerais [EMATER], 2004).

Traditionally, during ripening, Canastra cheeses are scraped to remove filamentous fungi that may grow on their surface, a practice known as “cheese toilette” (Leão et al., 2020). However, in recent years, some cheese makers have adopted the practice of preserving the autochthonous fungi grown during ripening, due to an interest in the sensory characteristics attributed to them, as well as the greater added value of the product. In 2020, a new regulation was approved in Minas Gerais that includes the conditions for AMC ripening, opening new market opportunities for the sector (Diário Oficial do Estado de Minas Gerais, 2020).

The bacterial microbiota of artisanal Canastra cheeses has already been investigated in several studies (Perin et al., 2017; Kamimura et al., 2020). However, its mycobiota is less well characterized in that, although several studies have focused on the characterization of fungi from Canastra cheeses using cultivation-dependent techniques (Borelli et al., 2006; Aragão, 2018; de Souza et al., 2021), Next Generation Sequencing (NGS)-based analyses remains rare. Notably, one such study demonstrated the predominance of *Diutina catenulata, Trichosporon* sp. and *Kodamaea ohmeri* in cheeses produced in the region (Kothe et al., 2022).

In this work, the characterization of the mycobiota of artisanal Canastra cheeses produced in the 9 cities that make up the region, was carried out, using ITS marker gene sequencing. To the best of our knowledge, this is the first work that included all the cities producing Canastra cheese and which identified the mycobiota core of the cheeses produced in wet and dry season. As many other studies have shown, fungi play a very important role for the nutrient-, organoleptic- and safety profile of cheeses and fermented foods in general. Therefore, this study was designed to understand the factors that influence the mycobiota of Canastra cheeses, with a particular focus on seasonality.

## 2. Materials and methods

### 2.1. Sample collection and experimental design

**FIGURE 1:**
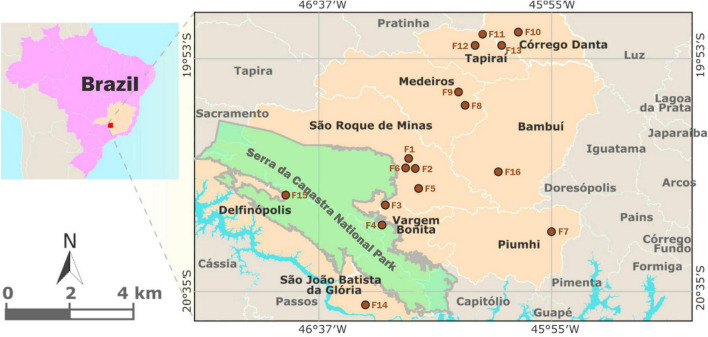
Cities involved in the production of Canastra cheese, Minas Gerais State, Brazil (cities area in beige color). Brown spots indicate the farms where the cheese samples were collected.

A total of 96 artisanal cheeses were collected from 16 different farms (3 cheeses per farm), over two periods (dry and wet seasons), in 9 cities in the Canastra region: Bambuí, Córrego D’Anta, Delfinópolis, Medeiros, Piumhi, São Roque de Minas, São Batista da Glória, Vargem Bonita and Tapiraí (Figure 1). All cheeses were produced with raw milk, industrial rennet, an endogenous ferment and salt. Information about the producers is presented in Table 1. The samples were stored in sterile bags and transported in thermal boxes to the Microbiology of Fermented Products Laboratory (FERMICRO) at the Microbiology Department of Universidade Federal de Viçosa (UFV) for further analysis.

**TABLE 1:** Metadata for the 16 Canastra cheese’s producers where the samples were collected.

Farm	City	Altitude
F1	São Roque de Minas	716 m
F2	São Roque de Minas	826 m
F3	Vargem Bonita	814 m
F4	Vargem Bonita	1,136 m
F5	São Roque de Minas	864 m
F6	São Roque de Minas	870 m
F7	Piumhi	784 m
F8	Medeiros	824 m
F9	Medeiros	840 m
F10	Córrego D’anta	713 m
F11	Tapiraí	950 m
F12	Medeiros	1,131 m
F13	Tapiraí	724 m
F14	S. João Batista do Glória	808 m
F15	Delfinópolis	741 m
F16	Bambuí	797 m

### 2.2. DNA extraction and library preparation

The DNA extraction was performed as following: cheese rinds were scraped to sample fungal cells and 400 mg of these scrapings were transferred to tubes containing ceramic pearls and 700 μL of lysis buffer (kit NucleoSpin Soil, Macherey-Nagel, Germany). The mixture was then lysed at 4,000 rpm for 1 minute in a tissue homogenizer (Precellys^®^, USA) and the following steps were provided as manufacturer’s recommendations. The extracted DNA quality was visualized on 0.8% agarose gel and quantified using a NanoDrop (Thermo Scientific NanoDrop^®^, USA). The fungal diversity in cheese rinds was analyzed by sequencing the amplified ITS region using ITS1F (5′-CTTGGTCATTTAGAGGAAGTAA-3′) and ITS2 primers (5′-GCTGCGTTCTTCATCGATGC-3′). For this, PCR was performed using 1 μl genomic DNA, 0.5 μl of each 10 μM primer, 5 μl of 5 × OneTaq Standard Reaction Buffer, 0.5 μl of 10 mM dNTPs and 0.63 units Taq polymerase (25 μl of final volume). PCR steps were: denaturation at 94°C/1 min; 94°C/30 sec, 52°C/30 sec and 68°C/30 sec (30 cycles); and a final extension step at 68°C/7 min. The products were quantified using a Qubit 4.0 fluorometer (Invitrogen) and reads were pooled in order to obtain a final concentration of 2 nM for each sample. The sequencing was performed by Macrogen (South Korea) in Illumina MiSeq platform (2 × 250 paired-end reads).

### 2.3. ITS analysis

Initially, the reads obtained from sequencing were demultiplexed and trimmed at the ends to remove the primers using the QIIME2^1^. QIIME2 was also used to remove singletons and chimeras, in addition to the assignment of Amplicon Sequence Variance (ASVs) and taxonomic annotation (Bolyen et al., 2019). The 18S and 5.8S flanking regions were removed using the ITSx (Bengtsson-Palme et al., 2013). Richness, evenness and alpha-diversity analyses were performed using Phyloseq package (McMurdie and Holmes, 2013) in R software (v. 4.1.2) (RStudio Team, 2020). Non-metric multidimensional scaling (NMDS) was obtained for beta diversity estimation using a counts-based Bray-Curtis dissimilarity measure using the vegan package (Oksanen et al., 2019). ANOSIM was used to determine significant differences in beta diversity based on the metadata collected. Shannon diversity index, inverse Simpson index and observed ASV’s were used to examine differences in alpha diversity.

## 3. Results

The fungal diversity of the rinds of Canastra cheeses was determined by amplicon sequencing of the ITS1 and ITS2 genes. Four samples (one from F1 – wet season; one from F1 – dry season; and two from F14 – dry season) were excluded due to the low quality of the sequencing reads. For the majority of the samples, the rarefaction curves reached the saturation plateau, demonstrating that the sequencing depth was sufficiently recovered.

**FIGURE 2:**
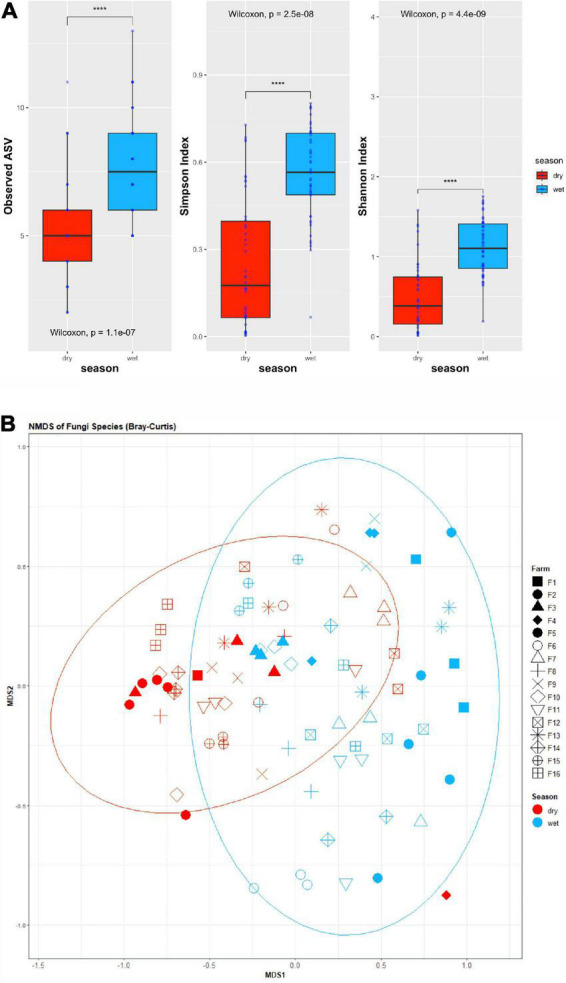
**(A)** Boxplots of alpha-diversity indices for the mycobiota of Canastra cheeses produced in wet and dry seasons. From left to right, the boxplots show the observed species (ASV’s), Simpsons diversity index and Shannon‘s diversity index of the cheeses grouped by season. Wilcoxon rank-sum test between wet and dry season illustrates significant differences in alpha diversity between seasons; **(B)** NMDS plot of the Bray-Curtis dissimilarity matrix of Canastra cheeses’ mycobiota at species level. Point colors indicate season (red points indicate dry season, blue points indicate wet season) and point shapes represent the various farms sampled. **** = *p*-value < 0.0001.

A higher diversity of fungal species was observed in cheese produced during the wet season (Figure 2A). Differences in both Shannon- and Simpson- evenness of the cheese mycobiota was observed between cities that make up the Canastra region. Both the richness and diversity of fungal species were affected by season in most cities, with lower richness and species occurring during the dry season. It is interesting to observe that in the dry season there was an increase in the richness of fungi in cheeses produced in Tapiraí, Bambui and Delfinópolis, as well as an increase in the number of species in cheese produced in Bambui and São Roque de Minas. Analysis of similarity matrix (ANOSIM) suggested that the cheeses’ mycobiota differs according to the producer (ANOSIM R = 0.21, *p* = 0.001), season (ANOSIM R = 0.18, *p* = 0.001) and city (ANOSIM R = 0.11, *p* = 0.008). However, no statistical differences were observed for altitude (ANOSIM R = 0.02, *p* = 0.194). The NMDS plot depicts the differences in the beta diversity of samples in a manner that reflects seasonal affects (Figure 2B).

**FIGURE 3:**
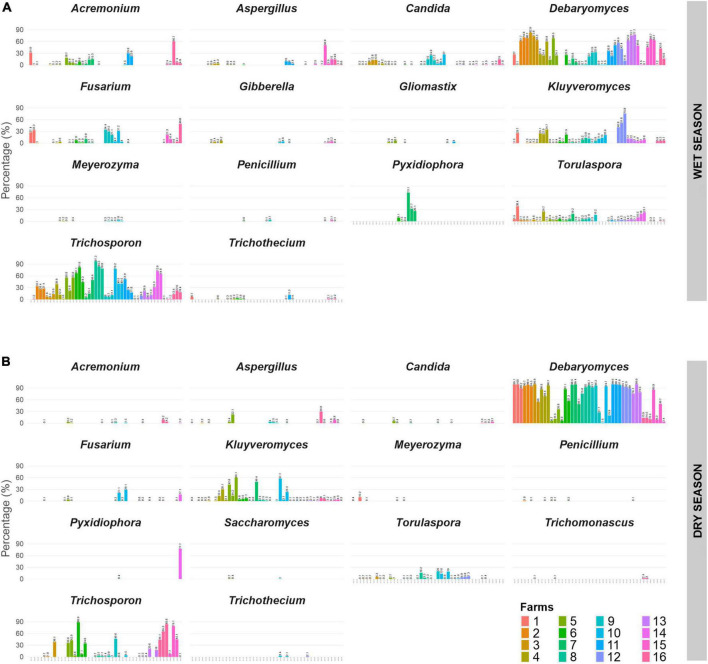
Relative abundance of the predominant fungi genera in Canastra cheeses produced during the wet season **(A)** and the dry season **(B)**, from 16 cheese-producing farms (indicated by different colors).

The main fungal genera identified by ITS analysis from farms in both seasons are shown in Figure 3. *Debaryomyces, Kluyveromyces, Torulaspora* and *Trichosporon* were detected in all farms evaluated, and thus they can play an important role in the cheese quality produced in Canastra region. In terms of filamentous fungi, *Fusarium, Acremonium* and *Aspergillus* were detected in most farms during the wet season (Figure 3A). However, their abundances were lower in the dry season (Figure 3B). *Penicillium*, a genus of filamentous fungus commonly associated with cheeses, was detected at relatively low abundances, during both wet and dry seasons. In addition to these, *Candida, Gibberella, Gliomastix, Meyerozyma, Pyxidiophora, Trichothecium* and *Trichomonascus* were among the genera identified in the samples evaluated.

**FIGURE 4:**
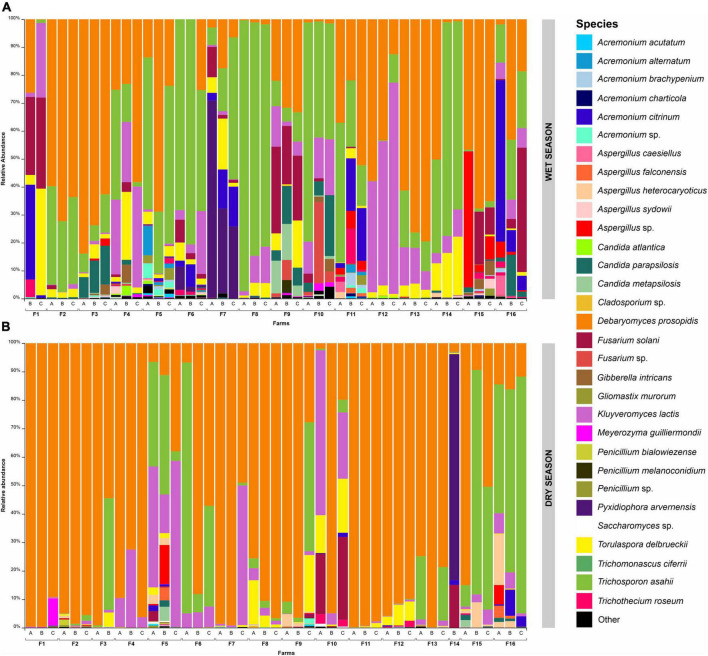
Barplot showing the relative abundance of the 25 main fungi species (ITS1 and ITS2) detected in the surface of Canastra cheese samples collected in wet season **(A)** and dry season **(B)**. Capital letters A, B, and C correspond to the cheese samples collected in the farms (from F1 to F16).

At the species level, *Debaryomyces prosopidis* was most abundant in most samples for both seasons (Figure 4). During the wet season (Figure 4A), *Trichosporon asahii, Kluyveromyces lactis*; *Fusarium solani*, a filamentous fungus, were the next most abundant species, respectively, followed by *Torulaspora delbrueckii* and *Acremonium citrinum*, another filamentous fungus. Several samples had a higher abundance of yeast, particularly *D. prosopidis.* F3-A (83.9%), F13-C (79.5%) and F13-B (76.3%) were notable for a high abundance of *D. prosopidis*. Samples F8-A, F8-B and F6-B showed the highest relative abundances of *T. asahii* (97.0, 83.5, and 80.6%, respectively). *K. lactis* was more abundant in samples F12-A (75.6%), F12-B (51.7%) and F12-C (39.7%), all collected on the same farm (F12, located in the city of Medeiros); and *T. delbrueckii* showed the highest abundances in samples F1-C (38.2%), F4-B (24.3%) and F14-C (20.9%). Regarding filamentous fungi, *F. solani* was more abundant in samples F1-C (44.5%) and F16-C (32.6%); *A. citrinum* showed the highest relative abundances in samples F16-A (57.8%), F1-B (33.9%) and F11-C (18.9%).

Similar species profiles were also observed in the dry season, although for those samples *Aspergillus heterocaryoticus* was among the 5 most abundant species (Figure 4B). Furthermore, there is a greater abundance of *D. prosopidis* in the dry season compared to the wet season. Relative abundance of this yeast at greater than 99.0% was observed for 7 samples collected in this season: F1-A (99.8%), F1-B (99.7%), F3-A (99.7%), F7-B (99.6%), F11-A (99.3%), F11-B (99.2%) and F13-B (99.1%). The highest relative abundances of *T. asahii* were observed in samples F6-A (88.1%), F15-B (83.1%) and F16-C (78.9%). F5-C, F7-C and F10-A were the samples that contained the highest abundances of *K. lactis* (58.2%, 58.0% and 49.0%, respectively). Lower abundance of *T. delbrueckii* was observed in samples F9-C (19.8%), F10-C (19.0%) and F8-A (15.5%). Finally, *A. heterocaryoticus* was the only filamentous fungus that was among the highest abundances observed for the dry season, with emphasis on samples F16-A (17.8%), F15-B (8.9%) and F9-A (4.3%).

## 4. Discussion

In recent years, the regulations relating to the production and marketing of ripened artisanal cheeses in Brazil have changed (Diário Oficial do Estado de Minas Gerais, 2020), bringing new opportunities for the cheese sector. However, few studies have been devoted to the identification of the mycobiota of artisanal cheese produced in the country (de Souza et al., 2021; Kothe et al., 2022). The characterization of the main groups of fungi that grow on the surface of cheese during the ripening process in different conditions is essential for understanding how seasonal and geographic aspects impact the cheese mycobiota and their influence role in product quality (de Souza et al., 2021). In this work, we observed that season significantly affected the mycobiota of Canastra cheeses (ANOSIM R = 0.18, *p* = 0.001), with higher richness and diversity values for the wet season (Figure 2A). In this region, the wet season extends from November to March, with average rainfall ranging from 208 to 313 mm/month. In the dry season, from April to October, the averages range from 18 to 67 mm/month. December has the highest relative humidity (80%) with August having the lowest (56%). The average temperature, however, does not vary greatly between seasons, with monthly averages ranging from 21.3 to 22.3°C for the wet season and 17.5 to 22.1°C for the dry season (Climate Data, 2022). Thus, the significant differences observed for the mycobiota of the cheese samples between wet and dry seasons is likely to be a result of the variation in rainfall and humidity.

Ultimately, seasonality was found to be the second most important factor in terms of its effect on the mycobiota (ANOSIM R = 0.18, *p* = 0.001). The biggest factor affecting the mycobiota was the farm of origin (ANOSIM R = 0.21, *p* = 0.001). Farm-associated differences likely reflect the different production approaches/trade secrets that are deployed by individual producers, including many other factors such as the quality of the raw material, the production environment, the intention to produce surface mold-ripened cheeses or just the traditional Canastra cheese-type, among others. Geographical location (ANOSIM R = 0.11, *p* = 0.008) of the cities was also important for artisanal Canastra cheeses. Altitude did not significantly impact the mycobiota (ANOSIM R = 0.02, *p* = 0.194). Thus, the season-dependent relative humidity, as well as the city of origin, and most importantly, the producer, influenced the Canastra mycbiota.

We also identified the mycobiota-associated taxa within Canastra cheeses that were common on all farms. These consisted of the yeast genera *Debaryomyces, Kluyveromyces, Torulaspora* and *Trichosporon*. Among the filamentous fungi, *Fusarium, Acremonium* and *Aspergillus* were the most abundant, although these have not been identified in all Canastra cheese producing farms. Both yeast and filamentous fungi play an important role in the sensory characteristics of artisanal cheese (Doğan and Tekiner, 2021; Penland et al., 2021). Yeasts are often found in artisanal cheese and can come from several sources in the production chain, such as raw milk, utensils, production environment and handlers (Lima et al., 2009; Quijada et al., 2018). In Brazil, *Candida catenulata, D. hansenii* and *T. delbrueckii* were the most abundant yeasts identified by culture-based techniques in Canastra cheeses after 5 days of ripening (Borelli et al., 2006). *D. hansenii* and *Kodamaea ohmeri* were also identified in cheeses produced in Serra do Salitre, Minas Gerais State (Lima et al., 2009). In a study of artisanal cheese produced in Serro region over 60 days of ripening, 19 yeast species were identified, including *D. hansenii, K. ohmeri* and *Kluyveromyces marxianus* (Cardoso et al., 2015).

Although *Debaryomyces* was the predominant genus in cheese produced in both seasons, greater relative abundance was observed for the dry season (Figures 3B, 4B). Drier environments tend to favor the growth of *Debaryomyces*, given its ability to tolerate high concentrations of salt (Wolfe et al., 2014). Interestingly, farms where yeast was less abundant in the wet season (F6, F7, F8, F9, F10, and F11) showed a significant increase in the dry season. Although it was not evaluated in this study, given our specific focus on external factors that influenced mycobiota composition, the sensory profile of cheese produced in the dry season may change compared to those produced in the wet season, given the role that this genus of yeast plays in flavor (Gori et al., 2012). *Kluyveromyces* spp. corresponds to one of the most abundant yeasts in smear cheeses, including French Reblochon and Livarot (Mounier and Coton, 2022), and has been associated with acidification and coagulation processes (Atanassova et al., 2016) as well as the sensory profiles of some cheese-types (de Freitas et al., 2008; Price et al., 2014; Centeno et al., 2022). In this study, we detected higher abundance of *T. delbrueckii* in the wet season on farms F1 and F14, as well as those produced in the dry season on farm F10 (Figure 4). The genus is commonly associated with smear used in the production of smear cheeses, although in much lower abundances than other yeasts such as *D. hansenii, Candida* spp. and *K. lactis* (Mounier and Coton, 2022). Regarding the filamentous fungi, *Fusarium, Acremonium* and *Aspergillus* had the highest abundance observed in this study. In smear cheeses, *Fusarium domesticum* is recognized as a microorganism that can contribute to preventing surface adhesion problems (a property known as “anticollanti”), in addition to being used as a ripening culture (Mounier and Coton, 2022). Ripening chambers of artisanal cheeses have already been identified as probable source of *F. solani* (de Souza et al., 2021). It is important to highlight the mycotoxigenic potential of *Fusarium* capable of producing fumonisins and zearalenones (Crous et al., 2021). *Acremonium* stands out for being a fungus often related to problems in industrial cheeses (Kure and Skaar, 2019) and has been found in the air of the industrial production facilities of semi-hard cheeses (Kure et al., 2004). Among the filamentous fungi commonly found in cheeses, *Penicillium* and *Aspergillus* correspond to the most problematic genera for the quality and safety (Kure and Skaar, 2019). Although with lower abundances in relation to the other taxa detected, we observed species infrequently or previously not reported in artisanal cheeses, such as *Aspergillus caesiellus, Aspergillus falconensis, Aspergillus heterocaryoticus* and *Aspergillus sydowii* (Figure 4). In a study carried out in Brazil, de Souza et al. (2021) detected *Aspergillus flavus* and *Aspergillus niger*, with *A. flavus* isolates positive for the production of aflatoxins B1 and B2 (*in vitro*). However, *Aspergillus* do not always produce toxins, therefore further analyses is required to determine if the *Aspergillus* species uncovered in this study are problematic.

Market opportunities for artisanal surface mold-ripened cheeses have already been recognized by Canastra producers for some years. The perception that cheeses with fungi on the surface can differentiate the product among the cheese making farms and, thereby, add value has been noted previously. Canastra cheesemakers are also conscious of the lack of studies specifically focused on the production of artisanal cheeses ripened by fungi in the Canastra region, which ends up making it difficult to understand the role of these microorganisms in the quality and safety of the product (Arantes, 2018). Thus, the results presented in this study can contribute to a better understanding of the influence of the season, city and farm on the mycobiota of Canastra cheeses and, consequently, on the quality and safety of the product.

## 5. Conclusion

In this study, the mycobiota of artisanal cheeses produced in the Canastra region, Brazil, was characterized based on ITS marker gene analysis. The yeasts *D. prosopidis, T. asahii, K. lactis* and *T. delbrueckii* were the most prevalent genera across all of the farms. The Canastra mycobiota also revealed filamentous fungi such as *F. solani, A. citrinum, Aspergillus* spp. and *Penicillium* spp., although in lower abundances than those observed for yeasts. The mycobiota of Canastra cheeses differed according to the farm, the city in which they are produced, and by the season, with greater fungal diversity observed for cheeses produced in the wet season. The altitude of the farms did not impact the mycobiota of the cheeses. As farm of origin (producer) had the biggest impact on the cheese mycobiota, further studies teasing apart production approaches are merited to understand how the various approaches influence the mycobiota. As mentioned previously, this manuscript was focused on seasonality and other parameters will be the focus of other investigations by our team in the future.

Although, in the Canastra region, the use of starter or secondary cultures is not allowed, the manipulation of production conditions in order to favor the development of autochthonous fungi, while controlling the growth of mycotoxigenic strains, can represent an interesting opportunity for artisanal cheesemakers to diversify their production adding value to the product, whilst preserving the authenticity of Canastra cheese. This becomes especially interesting at this time, after the recent change in regulation regarding the conditions for the production and marketing of ripened cheeses in the region.

In conclusion, we anticipate that this first study to characterize the mycobiota of Canastra cheeses involving producers from all cities across the wet and dry seasons has provided important new insights into the importance of the presence of fungi in artisanal cheeses produced in Canastra region, and will lead to the exploration of new markets and to the establishment of measures to improve the quality and safety of artisanal surface mold-ripened cheeses produced in Brazil.

## Data availability statement

The data presented in this study are deposited in the European Nucleotide Archive (ENA) repository, accession number PRJEB57877.

## Author contributions

JM, CF, and JGS conceived the study and its experimental design. IC, CF, JGS, and JM collected the samples and provided microbiological analysis. MS collaborated with microbiological analysis. JMS, TV, JM, and JL performed the metagenomics analyses and data visualization. JM, JL, and PC wrote the manuscript. All authors contributed to the article and approved the submitted version.

## Footnotes

1
https://qiime2.org/
